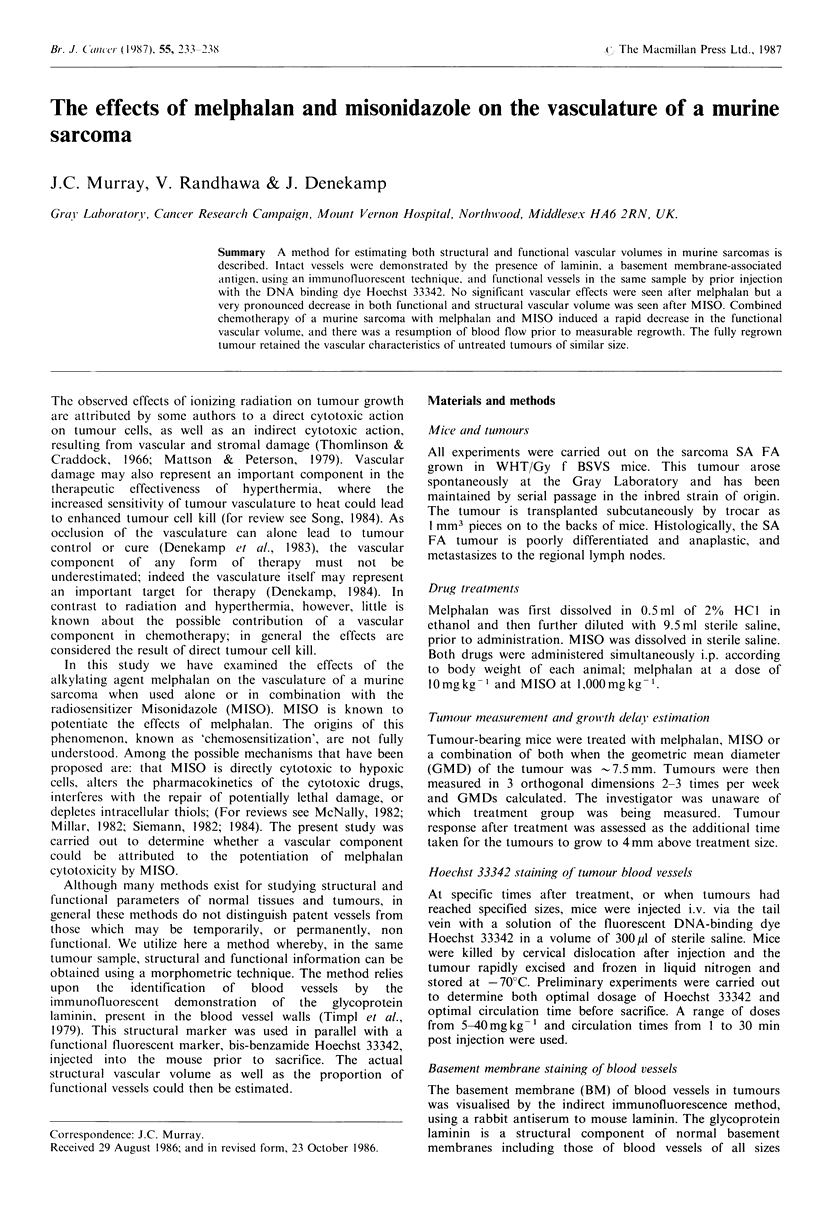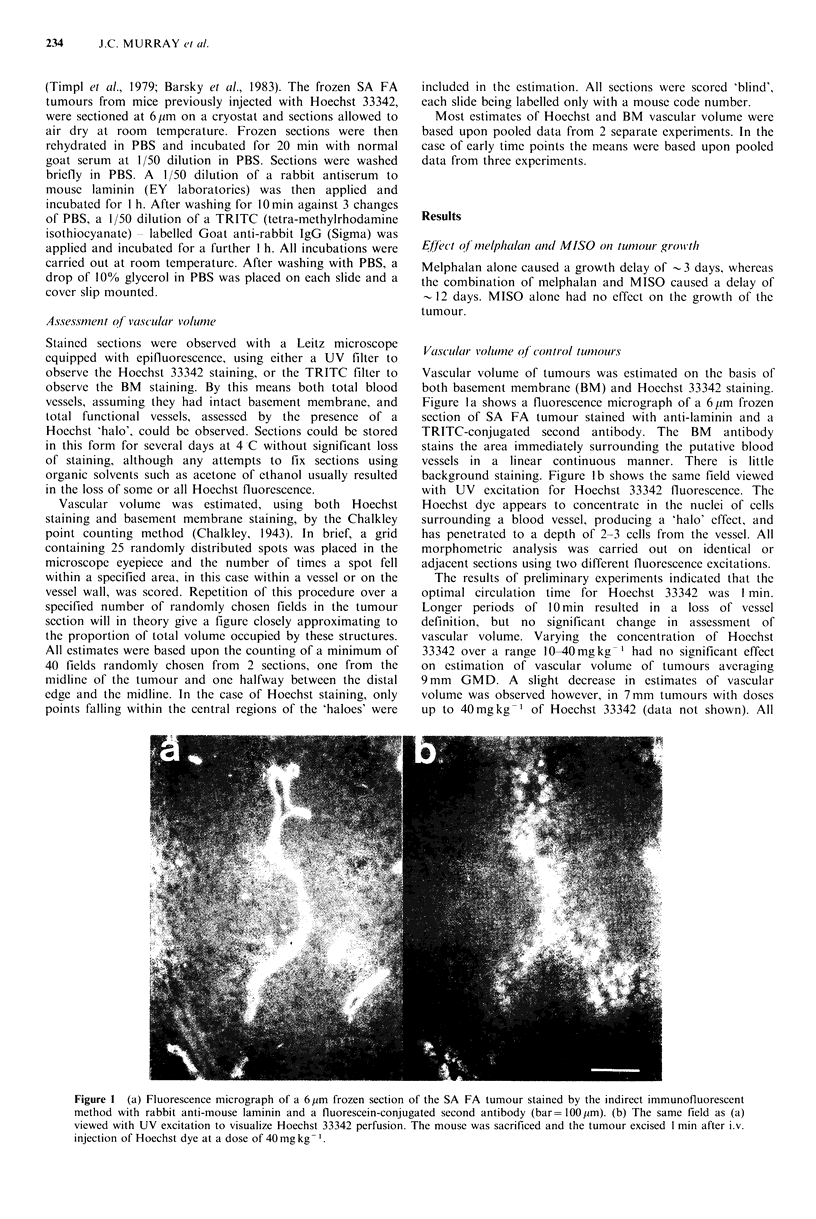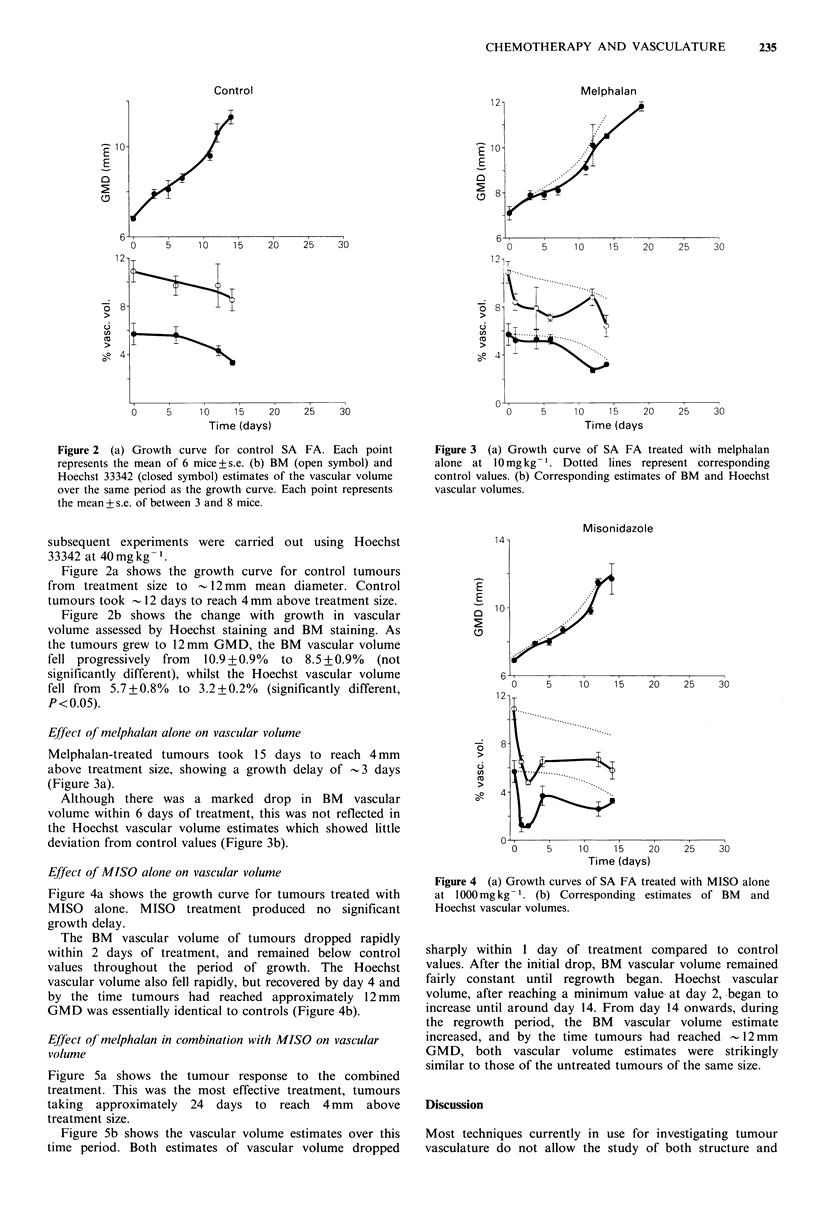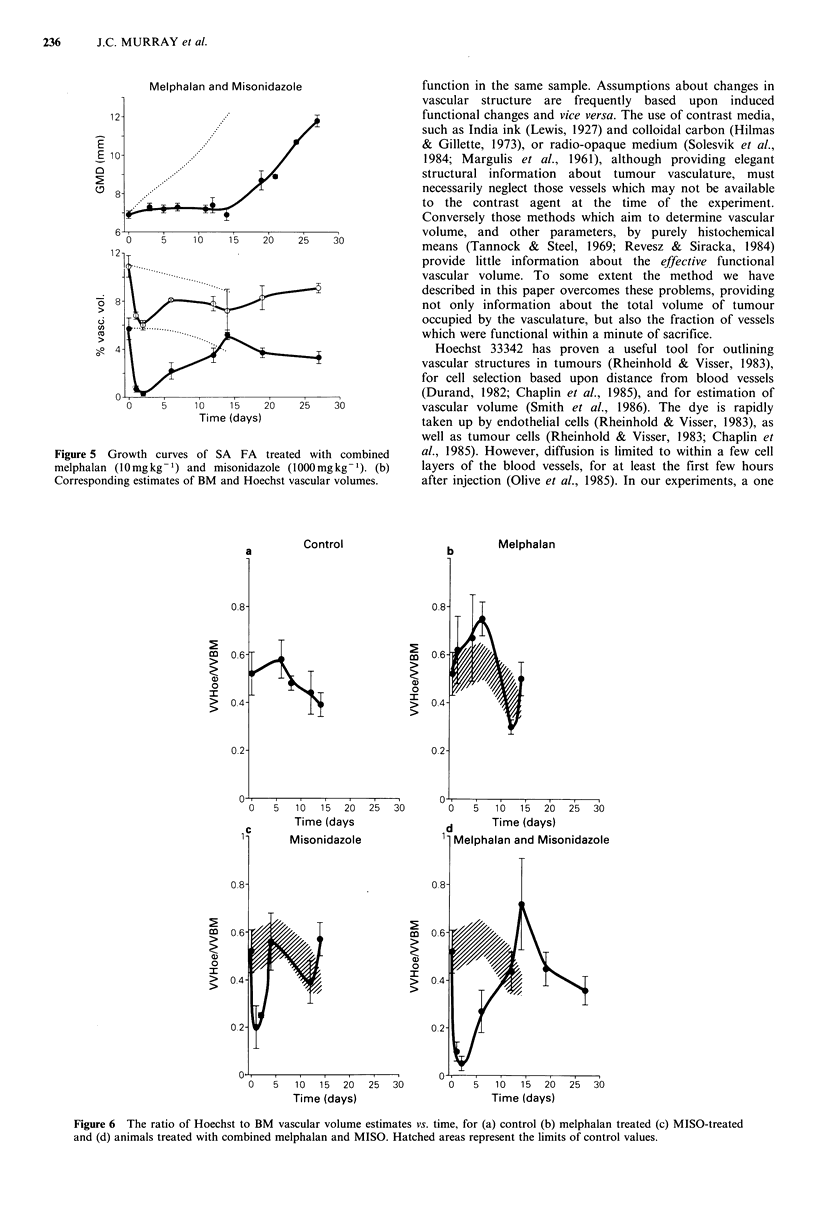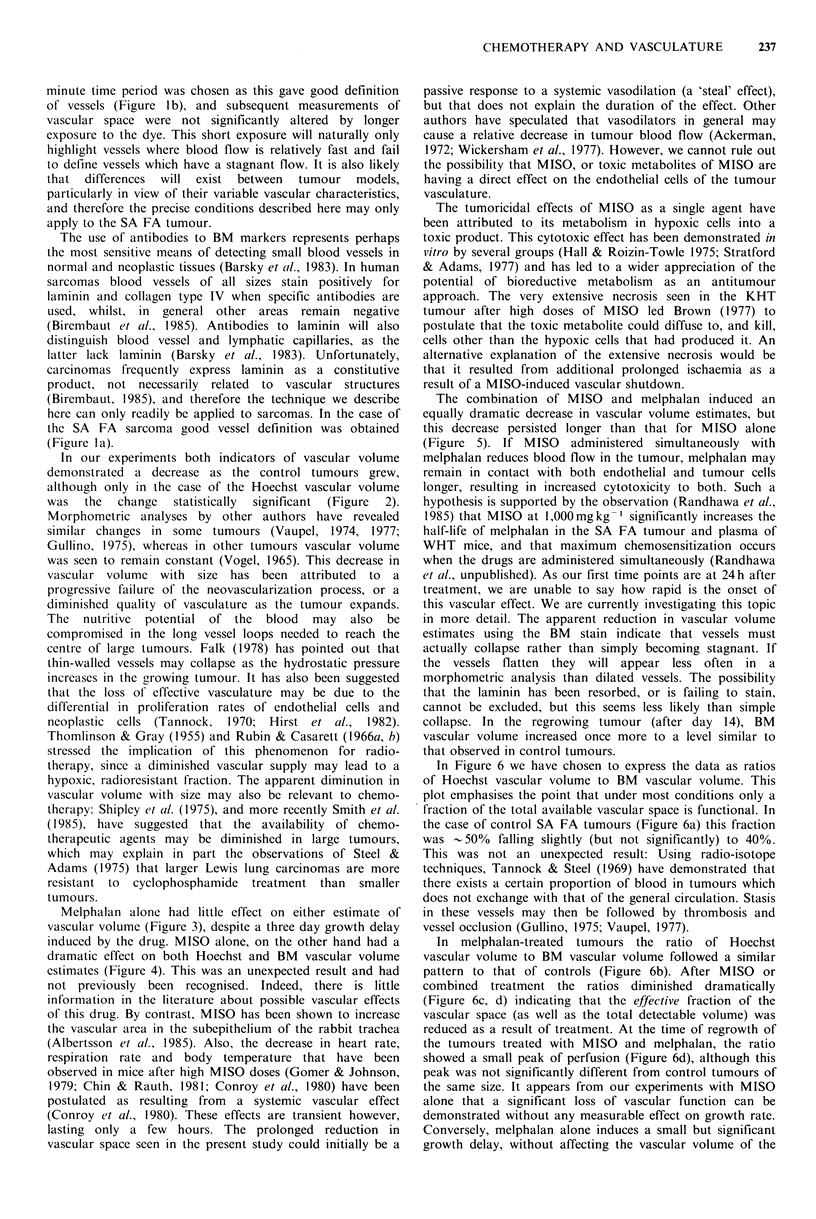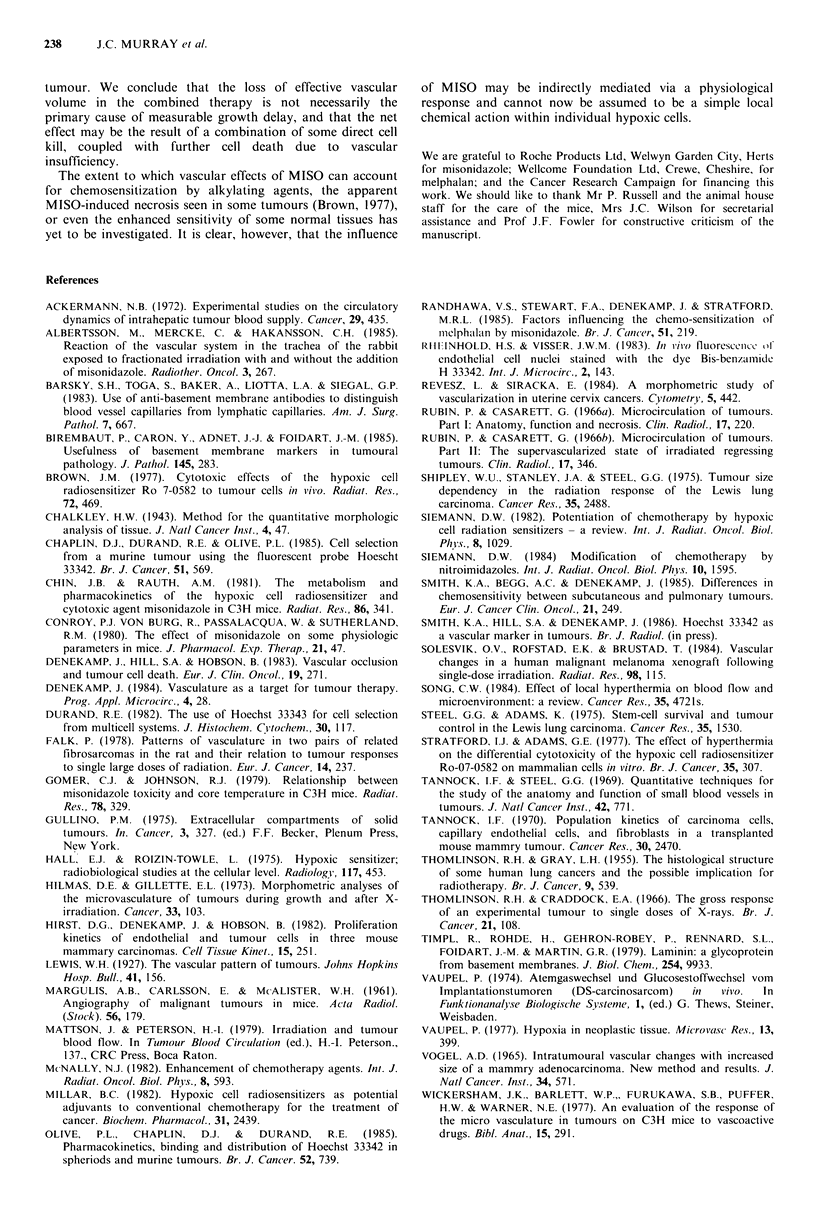# The effects of melphalan and misonidazole on the vasculature of a murine sarcoma.

**DOI:** 10.1038/bjc.1987.45

**Published:** 1987-03

**Authors:** J. C. Murray, V. Randhawa, J. Denekamp

## Abstract

**Images:**


Br. J. Cancer (1987), 55, 233–238 © The Macmillan Press Ltd., 1987

### The effects of melphalan and misonidazole on the vasculature of a murine sarcoma

J.C. Murray, V. Randhawa & J. Denekamp

*Gray Laboratory, Cancer Research Campaign, Mount Vernon Hospital, Northwood, Middlesex HA6 2RN, UK.*

**Summary** A method for estimating both structural and functional vascular volumes in murine sarcomas is described. Intact vessels were demonstrated by the presence of laminin, a basement membrane-associated antigen, using an immunofluorescent technique, and functional vessels in the same sample by prior injection with the DNA binding dye Hoechst 33342. No significant vascular effects were seen after melphalan but a very pronounced decrease in both functional and structural vascular volume was seen after MISO. Combined chemotherapy of a murine sarcoma with melphalan and MISO induced a rapid decrease in the functional vascular volume, and there was a resumption of blood flow prior to measurable regrowth. The fully regrown tumour retained the vascular characteristics of untreated tumours of similar size.

The observed effects of ionizing radiation on tumour growth are attributed by some authors to a direct cytotoxic action on tumour cells, as well as an indirect cytotoxic action, resulting from vascular and stromal damage (Thomlinson & Craddock, 1966; Mattson & Peterson, 1979). Vascular damage may also represent an important component in the therapeutic effectiveness of hyperthermia, where the increased sensitivity of tumour vasculature to heat could lead to enhanced tumour cell kill (for review see Song, 1984). As occlusion of the vasculature can alone lead to tumour control or cure (Denekamp *et al.*, 1983), the vascular component of any form of therapy must not be underestimated; indeed the vasculature itself may represent an important target for therapy (Denekamp, 1984). In contrast to radiation and hyperthermia, however, little is known about the possible contribution of a vascular component in chemotherapy; in general the effects are considered the result of direct tumour cell kill.

In this study we have examined the effects of the alkylating agent melphalan on the vasculature of a murine sarcoma when used alone or in combination with the radiosensitizer Misonidazole (MISO). MISO is known to potentiate the effects of melphalan. The origins of this phenomenon, known as 'chemosensitization', are not fully understood. Among the possible mechanisms that have been proposed are: that MISO is directly cytotoxic to hypoxic cells, alters the pharmacokinetics of the cytotoxic drugs, interferes with the repair of potentially lethal damage, or depletes intracellular thiols; (For reviews see McNally, 1982; Millar, 1982; Siemann, 1982; 1984). The present study was carried out to determine whether a vascular component could be attributed to the potentiation of melphalan cytotoxicity by MISO.

Although many methods exist for studying structural and functional parameters of normal tissues and tumours, in general these methods do not distinguish patent vessels from those which may be temporarily, or permanently, non functional. We utilize here a method whereby, in the same tumour sample, structural and functional information can be obtained using a morphometric technique. The method relies upon the identification of blood vessels by the immunofluorescent demonstration of the glycoprotein laminin, present in the blood vessel walls (Timpl *et al.*, 1979). This structural marker was used in parallel with a functional fluorescent marker, bis-benzamide Hoechst 33342, injected into the mouse prior to sacrifice. The actual structural vascular volume as well as the proportion of functional vessels could then be estimated.

Correspondence: J.C. Murray.
Received 29 August 1986; and in revised form, 23 October 1986.

### Materials and methods

### *Mice and tumours*

All experiments were carried out on the sarcoma SA FA grown in WHT/Gy f BSVS mice. This tumour arose spontaneously at the Gray Laboratory and has been maintained by serial passage in the inbred strain of origin. The tumour is transplanted subcutaneously by trocar as $1\,mm^3$ pieces on to the backs of mice. Histologically, the SA FA tumour is poorly differentiated and anaplastic, and metastasizes to the regional lymph nodes.

### *Drug treatments*

Melphalan was first dissolved in 0.5 ml of 2% HCl in ethanol and then further diluted with 9.5 ml sterile saline, prior to administration. MISO was dissolved in sterile saline. Both drugs were administered simultaneously i.p. according to body weight of each animal; melphalan at a dose of $10\,mg\,kg^{-1}$ and MISO at $1{,}000\,mg\,kg^{-1}$.

### *Tumour measurement and growth delay estimation*

Tumour-bearing mice were treated with melphalan, MISO or a combination of both when the geometric mean diameter (GMD) of the tumour was $\sim$7.5 mm. Tumours were then measured in 3 orthogonal dimensions 2–3 times per week and GMDs calculated. The investigator was unaware of which treatment group was being measured. Tumour response after treatment was assessed as the additional time taken for the tumours to grow to 4 mm above treatment size.

### *Hoechst 33342 staining of tumour blood vessels*

At specific times after treatment, or when tumours had reached specified sizes, mice were injected i.v. via the tail vein with a solution of the fluorescent DNA-binding dye Hoechst 33342 in a volume of 300 $\mu$l of sterile saline. Mice were killed by cervical dislocation after injection and the tumour rapidly excised and frozen in liquid nitrogen and stored at $-70$°C. Preliminary experiments were carried out to determine both optimal dosage of Hoechst 33342 and optimal circulation time before sacrifice. A range of doses from 5–40 $mg\,kg^{-1}$ and circulation times from 1 to 30 min post injection were used.

### *Basement membrane staining of blood vessels*

The basement membrane (BM) of blood vessels in tumours was visualised by the indirect immunofluorescence method, using a rabbit antiserum to mouse laminin. The glycoprotein laminin is a structural component of normal basement membranes including those of blood vessels of all sizes

(Timpl *et al.*, 1979; Barsky *et al.*, 1983). The frozen SA FA tumours from mice previously injected with Hoechst 33342, were sectioned at 6 $\mu$m on a cryostat and sections allowed to air dry at room temperature. Frozen sections were then rehydrated in PBS and incubated for 20 min with normal goat serum at 1/50 dilution in PBS. Sections were washed briefly in PBS. A 1/50 dilution of a rabbit antiserum to mouse laminin (EY laboratories) was then applied and incubated for 1 h. After washing for 10 min against 3 changes of PBS, a 1/50 dilution of a TRITC (tetra-methylrhodamine isothiocyanate) – labelled Goat anti-rabbit IgG (Sigma) was applied and incubated for a further 1 h. All incubations were carried out at room temperature. After washing with PBS, a drop of 10% glycerol in PBS was placed on each slide and a cover slip mounted.

*Assessment of vascular volume*

Stained sections were observed with a Leitz microscope equipped with epifluorescence, using either a UV filter to observe the Hoechst 33342 staining, or the TRITC filter to observe the BM staining. By this means both total blood vessels, assuming they had intact basement membrane, and total functional vessels, assessed by the presence of a Hoechst 'halo', could be observed. Sections could be stored in this form for several days at 4°C without significant loss of staining, although any attempts to fix sections using organic solvents such as acetone of ethanol usually resulted in the loss of some or all Hoechst fluorescence.

Vascular volume was estimated, using both Hoechst staining and basement membrane staining, by the Chalkley point counting method (Chalkley, 1943). In brief, a grid containing 25 randomly distributed spots was placed in the microscope eyepiece and the number of times a spot fell within a specified area, in this case within a vessel or on the vessel wall, was scored. Repetition of this procedure over a specified number of randomly chosen fields in the tumour section will in theory give a figure closely approximating to the proportion of total volume occupied by these structures. All estimates were based upon the counting of a minimum of 40 fields randomly chosen from 2 sections, one from the midline of the tumour and one halfway between the distal edge and the midline. In the case of Hoechst staining, only points falling within the central regions of the 'haloes' were included in the estimation. All sections were scored 'blind', each slide being labelled only with a mouse code number.

Most estimates of Hoechst and BM vascular volume were based upon pooled data from 2 separate experiments. In the case of early time points the means were based upon pooled data from three experiments.

### Results

*Effect of melphalan and MISO on tumour growth*

Melphalan alone caused a growth delay of ~3 days, whereas the combination of melphalan and MISO caused a delay of ~12 days. MISO alone had no effect on the growth of the tumour.

*Vascular volume of control tumours*

Vascular volume of tumours was estimated on the basis of both basement membrane (BM) and Hoechst 33342 staining. Figure 1a shows a fluorescence micrograph of a 6 $\mu$m frozen section of SA FA tumour stained with anti-laminin and a TRITC-conjugated second antibody. The BM antibody stains the area immediately surrounding the putative blood vessels in a linear continuous manner. There is little background staining. Figure 1b shows the same field viewed with UV excitation for Hoechst 33342 fluorescence. The Hoechst dye appears to concentrate in the nuclei of cells surrounding a blood vessel, producing a 'halo' effect, and has penetrated to a depth of 2–3 cells from the vessel. All morphometric analysis was carried out on identical or adjacent sections using two different fluorescence excitations.

The results of preliminary experiments indicated that the optimal circulation time for Hoechst 33342 was 1 min. Longer periods of 10 min resulted in a loss of vessel definition, but no significant change in assessment of vascular volume. Varying the concentration of Hoechst 33342 over a range 10–40 mg kg$^{-1}$ had no significant effect on estimation of vascular volume of tumours averaging 9 mm GMD. A slight decrease in estimates of vascular volume was observed however, in 7 mm tumours with doses up to 40 mg kg$^{-1}$ of Hoechst 33342 (data not shown). All

**Figure 1** (a) Fluorescence micrograph of a 6 $\mu$m frozen section of the SA FA tumour stained by the indirect immunofluorescent method with rabbit anti-mouse laminin and a fluorescein-conjugated second antibody (bar = 100 $\mu$m). (b) The same field as (a) viewed with UV excitation to visualize Hoechst 33342 perfusion. The mouse was sacrificed and the tumour excised 1 min after i.v. injection of Hoechst dye at a dose of 40 mg kg$^{-1}$.

**Figure 2** (a) Growth curve for control SA FA. Each point represents the mean of 6 mice±s.e. (b) BM (open symbol) and Hoechst 33342 (closed symbol) estimates of the vascular volume over the same period as the growth curve. Each point represents the mean±s.e. of between 3 and 8 mice.

subsequent experiments were carried out using Hoechst 33342 at $40\,mg\,kg^{-1}$.

Figure 2a shows the growth curve for control tumours from treatment size to ~12mm mean diameter. Control tumours took ~12 days to reach 4mm above treatment size.

Figure 2b shows the change with growth in vascular volume assessed by Hoechst staining and BM staining. As the tumours grew to 12mm GMD, the BM vascular volume fell progressively from 10.9±0.9% to 8.5±0.9% (not significantly different), whilst the Hoechst vascular volume fell from 5.7±0.8% to 3.2±0.2% (significantly different, $P<0.05$).

*Effect of melphalan alone on vascular volume*

Melphalan-treated tumours took 15 days to reach 4mm above treatment size, showing a growth delay of ~3 days (Figure 3a).

Although there was a marked drop in BM vascular volume within 6 days of treatment, this was not reflected in the Hoechst vascular volume estimates which showed little deviation from control values (Figure 3b).

*Effect of MISO alone on vascular volume*

Figure 4a shows the growth curve for tumours treated with MISO alone. MISO treatment produced no significant growth delay.

The BM vascular volume of tumours dropped rapidly within 2 days of treatment, and remained below control values throughout the period of growth. The Hoechst vascular volume also fell rapidly, but recovered by day 4 and by the time tumours had reached approximately 12mm GMD was essentially identical to controls (Figure 4b).

*Effect of melphalan in combination with MISO on vascular volume*

Figure 5a shows the tumour response to the combined treatment. This was the most effective treatment, tumours taking approximately 24 days to reach 4mm above treatment size.

Figure 5b shows the vascular volume estimates over this time period. Both estimates of vascular volume dropped

**Figure 3** (a) Growth curve of SA FA treated with melphalan alone at $10\,mg\,kg^{-1}$. Dotted lines represent corresponding control values. (b) Corresponding estimates of BM and Hoechst vascular volumes.

**Figure 4** (a) Growth curves of SA FA treated with MISO alone at $1000\,mg\,kg^{-1}$. (b) Corresponding estimates of BM and Hoechst vascular volumes.

sharply within 1 day of treatment compared to control values. After the initial drop, BM vascular volume remained fairly constant until regrowth began. Hoechst vascular volume, after reaching a minimum value at day 2, began to increase until around day 14. From day 14 onwards, during the regrowth period, the BM vascular volume estimate increased, and by the time tumours had reached ~12mm GMD, both vascular volume estimates were strikingly similar to those of the untreated tumours of the same size.

### Discussion

Most techniques currently in use for investigating tumour vasculature do not allow the study of both structure and

**Figure 5** Growth curves of SA FA treated with combined melphalan (10 mg $kg^{-1}$) and misonidazole (1000 mg $kg^{-1}$). (b) Corresponding estimates of BM and Hoechst vascular volumes.

function in the same sample. Assumptions about changes in vascular structure are frequently based upon induced functional changes and *vice versa*. The use of contrast media, such as India ink (Lewis, 1927) and colloidal carbon (Hilmas & Gillette, 1973), or radio-opaque medium (Solesvik *et al.*, 1984; Margulis *et al.*, 1961), although providing elegant structural information about tumour vasculature, must necessarily neglect those vessels which may not be available to the contrast agent at the time of the experiment. Conversely those methods which aim to determine vascular volume, and other parameters, by purely histochemical means (Tannock & Steel, 1969; Revesz & Siracka, 1984) provide little information about the *effective* functional vascular volume. To some extent the method we have described in this paper overcomes these problems, providing not only information about the total volume of tumour occupied by the vasculature, but also the fraction of vessels which were functional within a minute of sacrifice.

Hoechst 33342 has proven a useful tool for outlining vascular structures in tumours (Rheinhold & Visser, 1983), for cell selection based upon distance from blood vessels (Durand, 1982; Chaplin *et al.*, 1985), and for estimation of vascular volume (Smith *et al.*, 1986). The dye is rapidly taken up by endothelial cells (Rheinhold & Visser, 1983), as well as tumour cells (Rheinhold & Visser, 1983; Chaplin *et al.*, 1985). However, diffusion is limited to within a few cell layers of the blood vessels, for at least the first few hours after injection (Olive *et al.*, 1985). In our experiments, a one

**Figure 6** The ratio of Hoechst to BM vascular volume estimates *vs.* time, for (a) control (b) melphalan treated (c) MISO-treated and (d) animals treated with combined melphalan and MISO. Hatched areas represent the limits of control values.

minute time period was chosen as this gave good definition of vessels (Figure 1b), and subsequent measurements of vascular space were not significantly altered by longer exposure to the dye. This short exposure will naturally only highlight vessels where blood flow is relatively fast and fail to define vessels which have a stagnant flow. It is also likely that differences will exist between tumour models, particularly in view of their variable vascular characteristics, and therefore the precise conditions described here may only apply to the SA FA tumour.

The use of antibodies to BM markers represents perhaps the most sensitive means of detecting small blood vessels in normal and neoplastic tissues (Barsky *et al.*, 1983). In human sarcomas blood vessels of all sizes stain positively for laminin and collagen type IV when specific antibodies are used, whilst, in general other areas remain negative (Birembaut *et al.*, 1985). Antibodies to laminin will also distinguish blood vessel and lymphatic capillaries, as the latter lack laminin (Barsky *et al.*, 1983). Unfortunately, carcinomas frequently express laminin as a constitutive product, not necessarily related to vascular structures (Birembaut, 1985), and therefore the technique we describe here can only readily be applied to sarcomas. In the case of the SA FA sarcoma good vessel definition was obtained (Figure 1a).

In our experiments both indicators of vascular volume demonstrated a decrease as the control tumours grew, although only in the case of the Hoechst vascular volume was the change statistically significant (Figure 2). Morphometric analyses by other authors have revealed similar changes in some tumours (Vaupel, 1974, 1977; Gullino, 1975), whereas in other tumours vascular volume was seen to remain constant (Vogel, 1965). This decrease in vascular volume with size has been attributed to a progressive failure of the neovascularization process, or a diminished quality of vasculature as the tumour expands. The nutritive potential of the blood may also be compromised in the long vessel loops needed to reach the centre of large tumours. Falk (1978) has pointed out that thin-walled vessels may collapse as the hydrostatic pressure increases in the growing tumour. It has also been suggested that the loss of effective vasculature may be due to the differential in proliferation rates of endothelial cells and neoplastic cells (Tannock, 1970; Hirst *et al.*, 1982). Thomlinson & Gray (1955) and Rubin & Casarett (1966*a*, *b*) stressed the implication of this phenomenon for radiotherapy, since a diminished vascular supply may lead to a hypoxic, radioresistant fraction. The apparent diminution in vascular volume with size may also be relevant to chemotherapy: Shipley *et al.* (1975), and more recently Smith *et al.* (1985), have suggested that the availability of chemotherapeutic agents may be diminished in large tumours, which may explain in part the observations of Steel & Adams (1975) that larger Lewis lung carcinomas are more resistant to cyclophosphamide treatment than smaller tumours.

Melphalan alone had little effect on either estimate of vascular volume (Figure 3), despite a three day growth delay induced by the drug. MISO alone, on the other hand had a dramatic effect on both Hoechst and BM vascular volume estimates (Figure 4). This was an unexpected result and had not previously been recognised. Indeed, there is little information in the literature about possible vascular effects of this drug. By contrast, MISO has been shown to increase the vascular area in the subepithelium of the rabbit trachea (Albertsson *et al.*, 1985). Also, the decrease in heart rate, respiration rate and body temperature that have been observed in mice after high MISO doses (Gomer & Johnson, 1979; Chin & Rauth, 1981; Conroy *et al.*, 1980) have been postulated as resulting from a systemic vascular effect (Conroy *et al.*, 1980). These effects are transient however, lasting only a few hours. The prolonged reduction in vascular space seen in the present study could initially be a passive response to a systemic vasodilation (a 'steal' effect), but that does not explain the duration of the effect. Other authors have speculated that vasodilators in general may cause a relative decrease in tumour blood flow (Ackerman, 1972; Wickersham *et al.*, 1977). However, we cannot rule out the possibility that MISO, or toxic metabolites of MISO are having a direct effect on the endothelial cells of the tumour vasculature.

The tumoricidal effects of MISO as a single agent have been attributed to its metabolism in hypoxic cells into a toxic product. This cytotoxic effect has been demonstrated *in vitro* by several groups (Hall & Roizin-Towle 1975; Stratford & Adams, 1977) and has led to a wider appreciation of the potential of bioreductive metabolism as an antitumour approach. The very extensive necrosis seen in the KHT tumour after high doses of MISO led Brown (1977) to postulate that the toxic metabolite could diffuse to, and kill, cells other than the hypoxic cells that had produced it. An alternative explanation of the extensive necrosis would be that it resulted from additional prolonged ischaemia as a result of a MISO-induced vascular shutdown.

The combination of MISO and melphalan induced an equally dramatic decrease in vascular volume estimates, but this decrease persisted longer than that for MISO alone (Figure 5). If MISO administered simultaneously with melphalan reduces blood flow in the tumour, melphalan may remain in contact with both endothelial and tumour cells longer, resulting in increased cytotoxicity to both. Such a hypothesis is supported by the observation (Randhawa *et al.*, 1985) that MISO at 1,000 mg kg$^{-1}$ significantly increases the half-life of melphalan in the SA FA tumour and plasma of WHT mice, and that maximum chemosensitization occurs when the drugs are administered simultaneously (Randhawa *et al.*, unpublished). As our first time points are at 24 h after treatment, we are unable to say how rapid is the onset of this vascular effect. We are currently investigating this topic in more detail. The apparent reduction in vascular volume estimates using the BM stain indicate that vessels must actually collapse rather than simply becoming stagnant. If the vessels flatten they will appear less often in a morphometric analysis than dilated vessels. The possibility that the laminin has been resorbed, or is failing to stain, cannot be excluded, but this seems less likely than simple collapse. In the regrowing tumour (after day 14), BM vascular volume increased once more to a level similar to that observed in control tumours.

In Figure 6 we have chosen to express the data as ratios of Hoechst vascular volume to BM vascular volume. This plot emphasises the point that under most conditions only a fraction of the total available vascular space is functional. In the case of control SA FA tumours (Figure 6a) this fraction was ~50% falling slightly (but not significantly) to 40%. This was not an unexpected result: Using radio-isotope techniques, Tannock & Steel (1969) have demonstrated that there exists a certain proportion of blood in tumours which does not exchange with that of the general circulation. Stasis in these vessels may then be followed by thrombosis and vessel occlusion (Gullino, 1975; Vaupel, 1977).

In melphalan-treated tumours the ratio of Hoechst vascular volume to BM vascular volume followed a similar pattern to that of controls (Figure 6b). After MISO or combined treatment the ratios diminished dramatically (Figure 6c, d) indicating that the *effective* fraction of the vascular space (as well as the total detectable volume) was reduced as a result of treatment. At the time of regrowth of the tumours treated with MISO and melphalan, the ratio showed a small peak of perfusion (Figure 6d), although this peak was not significantly different from control tumours of the same size. It appears from our experiments with MISO alone that a significant loss of vascular function can be demonstrated without any measurable effect on growth rate. Conversely, melphalan alone induces a small but significant growth delay, without affecting the vascular volume of the tumour. We conclude that the loss of effective vascular volume in the combined therapy is not necessarily the primary cause of measurable growth delay, and that the net effect may be the result of a combination of some direct cell kill, coupled with further cell death due to vascular insufficiency.

The extent to which vascular effects of MISO can account for chemosensitization by alkylating agents, the apparent MISO-induced necrosis seen in some tumours (Brown, 1977), or even the enhanced sensitivity of some normal tissues has yet to be investigated. It is clear, however, that the influence of MISO may be indirectly mediated via a physiological response and cannot now be assumed to be a simple local chemical action within individual hypoxic cells.

We are grateful to Roche Products Ltd, Welwyn Garden City, Herts for misonidazole; Wellcome Foundation Ltd, Crewe, Cheshire, for melphalan; and the Cancer Research Campaign for financing this work. We should like to thank Mr P. Russell and the animal house staff for the care of the mice, Mrs J.C. Wilson for secretarial assistance and Prof J.F. Fowler for constructive criticism of the manuscript.

### References

ACKERMANN, N.B. (1972). Experimental studies on the circulatory dynamics of intrahepatic tumour blood supply. *Cancer*, **29**, 435.

ALBERTSSON, M., MERCKE, C. & HAKANSSON, C.H. (1985). Reaction of the vascular system in the trachea of the rabbit exposed to fractionated irradiation with and without the addition of misonidazole. *Radiother. Oncol.* **3**, 267.

BARSKY, S.H., TOGA, S., BAKER, A., LIOTTA, L.A. & SIEGAL, G.P. (1983). Use of anti-basement membrane antibodies to distinguish blood vessel capillaries from lymphatic capillaries. *Am. J. Surg. Pathol.* **7**, 667.

BIREMBAUT, P., CARON, Y., ADNET, J.-J. & FOIDART, J.-M. (1985). Usefulness of basement membrane markers in tumoural pathology. *J. Pathol.* **145**, 283.

BROWN, J.M. (1977). Cytotoxic effects of the hypoxic cell radiosensitizer Ro 7-0582 to tumour cells *in vivo*. *Radiat. Res.*, **72**, 469.

CHALKLEY, H.W. (1943). Method for the quantitative morphologic analysis of tissue. *J. Natl Cancer Inst.*, **4**, 47.

CHAPLIN, D.J., DURAND, R.E. & OLIVE, P.L. (1985). Cell selection from a murine tumour using the fluorescent probe Hoescht 33342. *Br. J. Cancer*, **51**, 569.

CHIN, J.B. & RAUTH, A.M. (1981). The metabolism and pharmacokinetics of the hypoxic cell radiosensitizer and cytotoxic agent misonidazole in C3H mice. *Radiat. Res.*, **86**, 341.

CONROY, P.J. VON BURG, R., PASSALACQUA, W. & SUTHERLAND, R.M. (1980). The effect of misonidazole on some physiologic parameters in mice. *J. Pharmacol. Exp. Therap.*, **21**, 47.

DENEKAMP, J., HILL, S.A. & HOBSON, B. (1983). Vascular occlusion and tumour cell death. *Eur. J. Clin. Oncol.*, **19**, 271.

DENEKAMP, J. (1984). Vasculature as a target for tumour therapy. *Prog. Appl. Microcirc.*, **4**, 28.

DURAND, R.E. (1982). The use of Hoechst 33343 for cell selection from multicell systems. *J. Histochem. Cytochem.*, **30**, 117.

FALK, P. (1978). Patterns of vasculature in two pairs of related fibrosarcomas in the rat and their relation to tumour responses to single large doses of radiation. *Eur. J. Cancer*, **14**, 237.

GOMER, C.J. & JOHNSON, R.J. (1979). Relationship between misonidazole toxicity and core temperature in C3H mice. *Radiat. Res.*, **78**, 329.

GULLINO, P.M. (1975). Extracellular compartments of solid tumours. *In. Cancer*, **3**, 327. (ed.) F.F. Becker, Plenum Press, New York.

HALL, E.J. & ROIZIN-TOWLE, L. (1975). Hypoxic sensitizer; radiobiological studies at the cellular level. *Radiology*, **117**, 453.

HILMAS, D.E. & GILLETTE, E.L. (1973). Morphometric analyses of the microvasculature of tumours during growth and after X-irradiation. *Cancer*, **33**, 103.

HIRST, D.G., DENEKAMP, J. & HOBSON, B. (1982). Proliferation kinetics of endothelial and tumour cells in three mouse mammary carcinomas. *Cell Tissue Kinet.*, **15**, 251.

LEWIS, W.H. (1927). The vascular pattern of tumours. *Johns Hopkins Hosp. Bull.*, **41**, 156.

MARGULIS, A.B., CARLSSON, E. & McALISTER, W.H. (1961). Angiography of malignant tumours in mice. *Acta Radiol. (Stock)*. **56**, 179.

MATTSON, J. & PETERSON, H.-I. (1979). Irradiation and tumour blood flow. In *Tumour Blood Circulation* (ed.), H.-I. Peterson., 137., CRC Press, Boca Raton.

McNALLY, N.J. (1982). Enhancement of chemotherapy agents. *Int. J. Radiat. Oncol. Biol. Phys.*, **8**, 593.

MILLAR, B.C. (1982). Hypoxic cell radiosensitizers as potential adjuvants to conventional chemotherapy for the treatment of cancer. *Biochem. Pharmacol.*, **31**, 2439.

OLIVE, P.L., CHAPLIN, D.J. & DURAND, R.E. (1985). Pharmacokinetics, binding and distribution of Hoechst 33342 in spheriods and murine tumours. *Br. J. Cancer*. **52**, 739.

RANDHAWA, V.S., STEWART, F.A., DENEKAMP, J. & STRATFORD, M.R.L. (1985). Factors influencing the chemo-sensitization of melphalan by misonidazole. *Br. J. Cancer*, **51**, 219.

RHEINHOLD, H.S. & VISSER, J.W.M. (1983). *In vivo* fluorescence of endothelial cell nuclei stained with the dye Bis-benzamide H 33342. *Int. J. Microcirc.*, **2**, 143.

REVESZ, L. & SIRACKA, E. (1984). A morphometric study of vascularization in uterine cervix cancers. *Cytometry*, **5**, 442.

RUBIN, P. & CASARETT, G. (1966*a*). Microcirculation of tumours. Part I: Anatomy, function and necrosis. *Clin. Radiol.*, **17**, 220.

RUBIN, P. & CASARETT, G. (1966*b*). Microcirculation of tumours. Part II: The supervascularized state of irradiated regressing tumours. *Clin. Radiol.*, **17**, 346.

SHIPLEY, W.U., STANLEY, J.A. & STEEL, G.G. (1975). Tumour size dependency in the radiation response of the Lewis lung carcinoma. *Cancer Res.*, **35**, 2488.

SIEMANN, D.W. (1982). Potentiation of chemotherapy by hypoxic cell radiation sensitizers – a review. *Int. J. Radiat. Oncol. Biol. Phys.*, **8**, 1029.

SIEMANN, D.W. (1984) Modification of chemotherapy by nitroimidazoles. *Int. J. Radiat. Oncol. Biol. Phys.* **10**, 1595.

SMITH, K.A., BEGG, A.C. & DENEKAMP, J. (1985). Differences in chemosensitivity between subcutaneous and pulmonary tumours. *Eur. J. Cancer Clin. Oncol.*, **21**, 249.

SMITH, K.A., HILL, S.A. & DENEKAMP, J. (1986). Hoechst 33342 as a vascular marker in tumours. *Br. J. Radiol.* (in press).

SOLESVIK, O.V., ROFSTAD, E.K. & BRUSTAD, T. (1984). Vascular changes in a human malignant melanoma xenograft following single-dose irradiation. *Radiat. Res.*, **98**, 115.

SONG, C.W. (1984). Effect of local hyperthermia on blood flow and microenvironment: a review. *Cancer Res.*, **35**, 4721s.

STEEL, G.G. & ADAMS, K. (1975). Stem-cell survival and tumour control in the Lewis lung carcinoma. *Cancer Res.*, **35**, 1530.

STRATFORD, I.J. & ADAMS, G.E. (1977). The effect of hyperthermia on the differential cytotoxicity of the hypoxic cell radiosensitizer Ro-07-0582 on mammalian cells *in vitro*. *Br. J. Cancer*, **35**, 307.

TANNOCK, I.F. & STEEL, G.G. (1969). Quantitative techniques for the study of the anatomy and function of small blood vessels in tumours. *J. Natl Cancer Inst.*, **42**, 771.

TANNOCK, I.F. (1970). Population kinetics of carcinoma cells, capillary endothelial cells, and fibroblasts in a transplanted mouse mammry tumour. *Cancer Res.*, **30**, 2470.

THOMLINSON, R.H. & GRAY, L.H. (1955). The histological structure of some human lung cancers and the possible implication for radiotherapy. *Br. J. Cancer*, **9**, 539.

THOMLINSON, R.H. & CRADDOCK, E.A. (1966). The gross response of an experimental tumour to single doses of X-rays. *Br. J. Cancer*, **21**, 108.

TIMPL, R., ROHDE, H., GEHRON-ROBEY, P., RENNARD, S.L., FOIDART, J.-M. & MARTIN, G.R. (1979). Laminin: a glycoprotein from basement membranes. *J. Biol. Chem.*, **254**, 9933.

VAUPEL, P. (1974). Atemgaswechsel und Glucosestoffwechsel vom Implantationstumoren (DS-carcinosarcom) *in vivo*. In *Funktionanalyse Biologische Systeme*, **1**, (ed.) G. Thews, Steiner, Weisbaden.

VAUPEL, P. (1977). Hypoxia in neoplastic tissue. *Microvasc Res.*, **13**, 399.

VOGEL, A.D. (1965). Intratumoural vascular changes with increased size of a mammry adenocarcinoma. New method and results. *J. Natl Cancer. Inst.*, **34**, 571.

WICKERSHAM, J.K., BARLETT, W.P.,, FURUKAWA, S.B., PUFFER, H.W. & WARNER, N.E. (1977). An evaluation of the response of the micro vasculature in tumours on C3H mice to vascoactive drugs. *Bibl. Anat.*, **15**, 291.